# Supplementary material for: Rapid and Nondestructive Evaluation of Wheat Chlorophyll under Drought Stress Using Hyperspectral Imaging
Source: Int J Mol Sci. 2023 Mar 18;24(6):5825. doi: 10.3390/ijms24065825 (PMC10056805; doi:10.3390/ijms24065825)
Supplement: Supplementary file 1 [file ijms-24-05825-s001.zip › Supplementary Materials/Figure S1.The interaction of light with plant leaves.pdf]

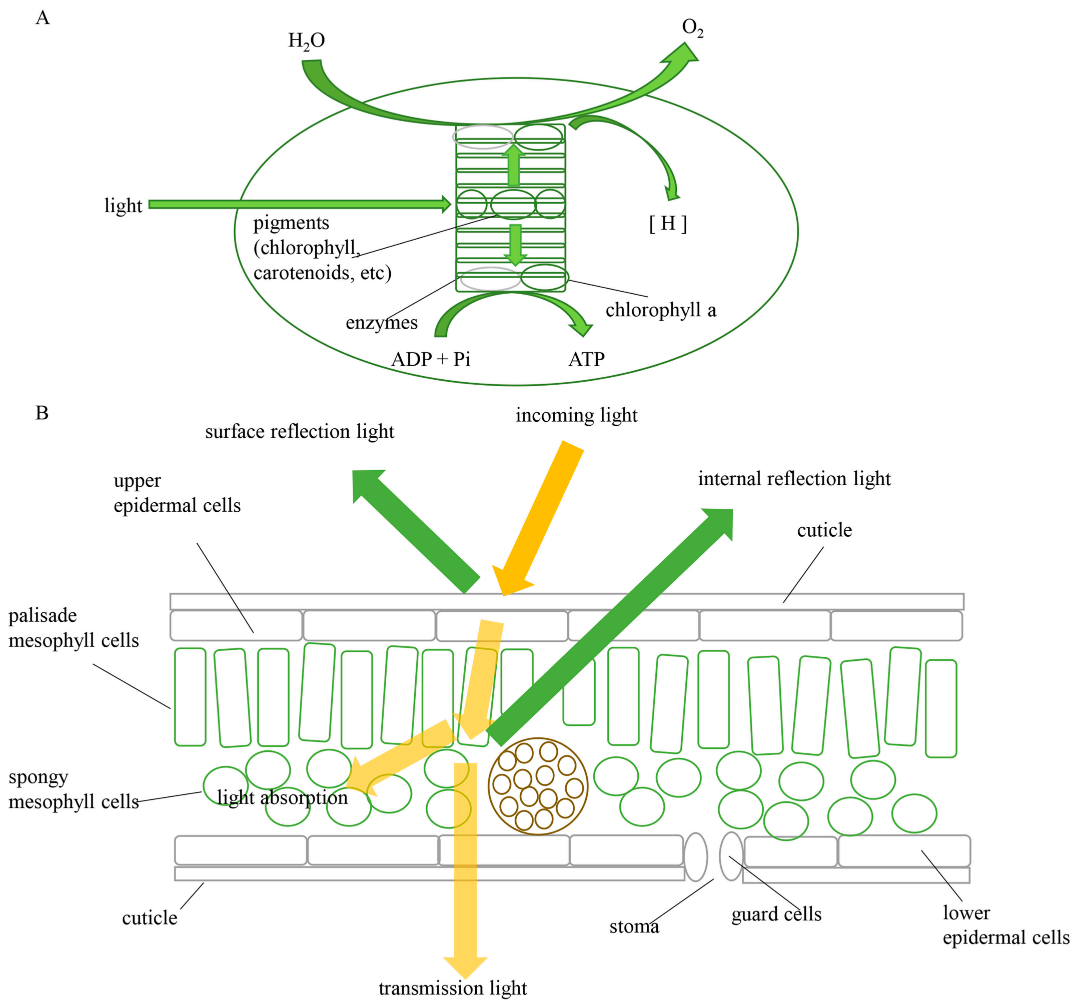

Figure S1. The interaction of light with plant leaves. (A) the mechanism of absorption light by chloroplast; (B) optical properties of leaves.
