# Supplementary material for: Rapid and Nondestructive Evaluation of Wheat Chlorophyll under Drought Stress Using Hyperspectral Imaging
Source: Int J Mol Sci. 2023 Mar 18;24(6):5825. doi: 10.3390/ijms24065825 (PMC10056805; doi:10.3390/ijms24065825)
Supplement: Supplementary file 1 [file ijms-24-05825-s001.zip › Supplementary Materials/Highlights.pdf]

Highlights：

1. Hyperspectral imaging is a rapid, nondestructive, technique for accurately estimating chlorophyll in wheat leaves.

2. Using wheat varieties with large genetic diversity and different treatments provides more representative results.

3. Characteristic bands associated with chlorophyll and drought stress were extracted using correlation analysis and machine learning algorithms.

4. The combination of spectrum and image characteristics (L*, a*, and b*) can improve estimation accuracy of chlorophyll content.

5. The technical method established in this study has great potential for evaluating chlorophyll content and stress resistance, as well as optimizing crop breeding.
