# Supplementary material for: Rapid and Nondestructive Evaluation of Wheat Chlorophyll under Drought Stress Using Hyperspectral Imaging
Source: Int J Mol Sci. 2023 Mar 18;24(6):5825. doi: 10.3390/ijms24065825 (PMC10056805; doi:10.3390/ijms24065825)
Supplement: Supplementary file 1 [file ijms-24-05825-s001.zip › Supplementary Materials/Table S1.Germplasm information of 335 wheat varieties (strains).pdf]

Table S1. Germplasm information of 335 wheat varieties (strains)

Numbers	Varieties (Strains)	Numbers	Varieties (Strains)
1	Fielder	169	Kenong199
2	Bobwhite	170	Ningmai13
3	Xinong979	171	Shimai12
4	Triticum dicoccum Schuebl	172	Zhongmai175
5	Triticum compactum Host	173	Zhouyuan9369
6	Triticum turgidum Linn	174	Zhoumai22
7	Triticum persicum Zhuk	175	Mianmai46
8	Foshou wheat	176	Ningmai15
9	Triticum turgidum subsp. durum (Desfontaines) Husnot	177	NingnuomaiNo.1
10	Triticum dicoccoides Korn	178	Xinong928
11	Triticale(4X)	179	Zhongyou206
12	TriticaleⅡ	180	Zhoumai23
13	Triticum turgidum subsp. durum (Desfontaines) Husnot 4286	181	JI5265
14	091	182	Shimai19
15	Brazil	183	ZhongjiaoNo.2
16	Mexico67	184	Xinmai26
17	Mexico61	185	Kaimai21
18	Mexico69	186	Jiaomai266
19	Indian Lanli	187	Nongda1108
20	15JH6 Hongli	188	XunongNo.7
21	Unknown (Hongli)	189	Zhongxin78
22	Anhong	190	Zhoumai26
23	JiheiNo.1	191	Bainong207
24	Unknown (Barly/Aegilops tauschii Coss//Triticum turgidum subsp. durum (Desfontaines) Husnot⊕-1-4-4-2-3-2-2-2)	192	Zhoumai32
25	Unknown(zh-wheat/Lang//zh-wheat-1-1-2-1-1)	193	Xinong109
26	Unknown (lang/Indian Lanli(3)-2-9-1-2-1-4-1-1-1)	194	Xinong511
27	Baibanaihua1	195	Chang6794
28	Baibanaihua2	196	TongmaiNo.6
29	Lanli(H)	197	Xinong100
30	Hongli(h)	198	Zhongmai578
31	Zisemai	199	WeimaiNo.9
32	Nuomai	200	Jimai22
33	IR4	201	Jimai22MYBmutant
34	IR145	202	UNIVERSALⅡ
35	IR150	203	SUNSET
36	IR165	204	JOHNBROWN
37	IR166	205	RED-BOBS-222

38	IR170	206	PUSA80-5
39	IR171	207	ALSHVEDE
40	IR176	208	LAGODEKHIS-GRDZELTAVT AVA
41	IR178	209	KENNEDYHARLANJ.R38
42	IR179	210	MEX94.10.1
43	IR181	211	QRO94.1.1
44	IR182	212	TOL93.1.2
45	IR183	213	GHARFLOR-1611
46	IR185	214	LOHARIY91-92NO.123
47	IR188	215	GRIFONE235
48	IR226	216	MONTEZUMA-CLUB
49	IR228	217	LAGEADINHO
50	IR236	218	SENMARQ
51	IR39	219	INDIANOMOCHO6296
52	IR44	220	ABYSSINIAN43
53	IR74	221	RASHKOOL
54	IR79	222	TIGARIA
55	IR89	223	GEHUNFARAMI
56	IR192	224	NEPHADINDIAN
57	IR193	225	AURORE4323A
58	IR196	226	HOMARANO44
59	IR197	227	2794:AE
60	IR198	228	Samson
61	IR202	229	FS056
62	IR209	230	9H65A
63	IR211	231	RAVENNA
64	IR214	232	ENESCO
65	IR216	233	SIRMIONE
66	IR218	234	Neijiang31
67	IR220	235	Dong2-8
68	IR247	236	XingmaiNo.1
69	BimaNo.4	237	Xian83
70	Wuyiwheat	238	Zaosui30
71	Sanyuanwheat	239	Nanda96co76
72	Xibei612	240	Changwei18
73	WannianNo.2	241	Zhengmai9201
74	ShannongNo.1	242	PRIMOASI
75	Shijiazhuang54	243	WeimaiNo.6
76	JinanNo.4	244	ShuangjiNo.4
77	XuzhouNo.8	245	PANDAS
78	Baiyoubao	246	ZhongjiaoNo.1
79	Kequan	247	Colotana
80	Xiannong39	248	Camacrania
81	FengchanNo.1	249	Mianyang79-2
82	BeijingNo.10	250	Mexifen
83	HanxuanNo.10	251	Xifeng
84	JinanNo.8	252	Heibao
85	JinanNo.9	253	H798
86	EmaiNo.6	254	Claudia

87	FengchanNo.3	255	Hongmangyouzimai
88	YangmaiNo.1	256	Baiyoumai
89	QingchunNo.5	257	Chuan84-7045
90	TaishanNo.1	258	Tupatecor
91	XiaoyanNo.4	259	ACForemost
92	YangmaiNo.2	260	Penawa
93	JimaiNo.3	261	Owens
94	Keyi26	262	Tosson
95	MachangNo.2	263	KehongNo.1
96	Wan7107	264	Saet
97	JimaiNo.6	265	Ourou
98	LongchunNo.8	266	ACvista
99	YanshiNo.4	267	Abo
100	QingfengNo.1	268	Gezhoumai
101	Yannong15	269	Xiaoziganzi
102	WeimaiNo.4	270	Jichun1016
103	FengkangNo.7	271	Baixiaomai
104	Xiannong151	272	Chayazheda29
105	Yumai18	273	Sanyuehuang
106	AimengniuⅣ	274	Napo63
107	FengkangNo.8	275	Laser
108	QinmaiNo.1	276	Krac66
109	Jinan13	277	Baipu
110	Jimai22	278	Tumangmai
111	Jimai23	279	Duiker
112	Xichang76-9	280	Sifangmai
113	Xuzhou21	281	Heputou
114	Bainong791	282	Carpo
115	Jimai26	283	Glenlea
116	YumaiNo.10	284	Jingyang60
117	XianNo.8	285	Zaoyangmai
118	Yumai15	286	FRECCIA
119	Lumai15	287	SAGITTARIO
120	Neixiang182	288	Meiyuan1
121	Yumai19	289	Meiyuan4
122	Wanmai19	290	Meiyuan8
123	Xinong88	291	Meiyuan18
124	Xindong20	292	Meiyuan31
125	Shan229	293	Meiyuan44
126	Gaoyou503	294	Meiyuan72
127	Shi-4185	295	Meiyuan77
128	Wanmai31	296	Meiyuan87
129	Yumai54	297	Meiyuan291
130	ZhongmaiNo.9	298	Meiyuan294
131	K173	299	Meiyuan300
132	K193	300	Unknown (PASTOR-2/HUBARA-5)
133	K153	301	Unknown (MELLAL-1/OUEDZEM-1)

134	K163	302	Unknown (YEBROUD´S´//DOVE´S´/SERI/3/SAFI-1)
135	R59chuannong12	303	Unknown (MUNIA//CHEN/ALTAR84/3/CHEN/AEGILOPSSQUARROSA(TAUS)//BCN/4/MARCHOUCH-8)
136	Emai16	304	Unknown (KARAWAN-1/TALLO3//JADIDA-2)
137	Emai17	305	Unknown (TEVEE-1/STAR´S´/3/ACHTAR*3//KANZ/KS85-8-4)
138	Emai18	306	Unknown (MOUKA-4*2/4/KEA´S´/3/MN72252//HD2169/BOW´S´)
139	Huaimai20	307	4332a
140	MianyangNo.31	308	HeshengNo.2
141	Yannong22	309	MK95
142	JimaiNo.20	310	MK165
143	LiaomaiNo.16	311	MK186
144	Lunxuan987	312	PENNY
145	WeimaiNo.8	313	IRAGI
146	Yannong23	314	2821:AE
147	Yangmai13	315	2495:AE
148	Yangmai16	316	1748:AE
149	Xiaoyan22	317	MICH89.1.3
150	Zhoumai16	318	OAX93.1.1.1
151	Ningmai12	319	OSJAKOWSKI
152	Shiluan02-1	320	AimengniuⅤ
153	Shimai12	321	Baihuomai
154	Taishan23	322	ZhengzhouNo.5
155	Aikang58	323	Xuzhou25
156	JiningNo.16	324	Zhoumai20
157	LianmaiNo.12	325	GLUYASEARLY
158	Mianmai39	326	MARROCOS16.3608
159	Mianmai40	327	REDSPRING
160	Qinnong142	328	CENTEIROZ44
161	Shannong12	329	Yumai49
162	Wanmai50	330	Ningmai9
163	Xinong979	331	Yangmai12
164	Yangmai17	332	VAIOLET
165	Yumai949	333	Zhongluo08-3
166	ZhenmaiNo.6	334	KexinNo.9
167	Zhoumai18	335	Beijing0045
168	Jimai22		
