# Supplementary material for: Rapid and Nondestructive Evaluation of Wheat Chlorophyll under Drought Stress Using Hyperspectral Imaging
Source: Int J Mol Sci. 2023 Mar 18;24(6):5825. doi: 10.3390/ijms24065825 (PMC10056805; doi:10.3390/ijms24065825)
Supplement: Supplementary file 1 [file ijms-24-05825-s001.zip › Supplementary Materials/Table S2.SPAD values of wheat leaf under different soil moisture condition conditions.pdf]

Table S2. SPAD values of wheat leaf under different soil moisture condition conditions

Numbers	SPAD value (CK)	SPAD value (DS)	Numbers	SPAD value (CK)	SPAD value (DS)
1	57.09	55.4	169	59.14	54.73
2	54.66	49.45	170	61.59	57.62
3	56.77	54.07	171	43.55	47.82
4	41.09	41.76	172	51.37	59.17
5	52.08	49.54	173	53.84	58.85
6	47.16	49.32	174	55.58	56.08
7	40.09	27.63	175	58.92	47.88
8	53.22	10.31	176	59.93	57.81
9	38.33	50.12	177	58.54	59.31
10	51.31	40.15	178	57.47	51.2
11	54.14	60.4	179	54.63	60.57
12	49.25	39.35	180	60.77	58.82
13	60.96	37.2	181	56.05	59.73
14	60.31	47.28	182	55.68	55.92
15	57.3	55.28	183	51.1	53.52
16	49.21	38.61	184	48.55	48.13
17	57.03	47.87	185	52.01	56.03
18	51.7	13.33	186	51.53	51.01
19	50.94	41.09	187	45.49	54.05
20	54.77	53.98	188	58.31	57.96
21	60.52	58.52	189	50.31	52.35
22	55.84	42.89	190	43.76	54.21
23	52.99	51.97	191	46.77	59.36
24	47.91	55	192	48.49	53.37
25	60.58	63.4	193	51.45	56.06
26	56.12	21.9	194	48.34	52.59
27	56.1	57.94	195	50.05	52.83
28	67.58	59.59	196	51.81	54.68
29	50.83	50.37	197	54.11	54.89
30	56.85	48.23	198	54.67	62.77
31	58.37	58.51	199	41	54.72
32	57.23	58.61	200	48.46	57.91
33	54.31	57.6	201	40.79	51.95
34	52.34	44.53	202	53.79	58.29
35	51.39	50.92	203	42.9	25.85
36	52.73	55.5	204	44.83	48.29
37	36.58	47.4	205	46.39	18.4
38	52.32	53.29	206	44.15	53.37
39	51.06	55.56	207	42.1	47.94
40	53.39	47.86	208	48.16	39.79
41	45.57	52.27	209	52.1	37.51
42	47.93	54.6	210	27.15	52.71
43	53.85	51.75	211	41.13	46.15
44	55.66	51.22	212	41.73	29.35
45	52.94	56.39	213	50.75	30.97
46	55.81	58.6	214	46.91	39.84

47	50.5	44.69	215	44.27	46.92
48	55.13	49.6	216	43.32	40.43
49	46.53	52.06	217	50.11	46.5
50	52.71	52.24	218	48.26	38.61
51	55.41	55.31	219	56.47	53.31
52	55.27	53.96	220	67.31	46.91
53	46.99	53.88	221	38.15	35.46
54	53.11	51.2	222	42.39	46.35
55	49.55	48.79	223	49.67	45.84
56	52.23	53.2	224	45.18	44.43
57	44.25	50.28	225	40.01	49.88
58	47.41	49.51	226	40.24	44.43
59	53.87	55.07	227	43.05	50.27
60	52.11	49.5	228	54.77	51.97
61	51.45	55.4	229	50.96	59.96
62	51.86	58.67	230	35.3	53.97
63	52.55	52.68	231	51.49	54.36
64	51.21	50.69	232	58.59	64.01
65	47.18	44.4	233	44.42	53.95
66	48.87	46.1	234	43.82	43.26
67	52.8	43.58	235	51.15	57.79
68	50.87	52	236	42.62	51.65
69	52.94	55.04	237	52.67	47.73
70	44.37	42.28	238	46.93	60.67
71	49.5	34.28	239	45.98	52.65
72	46.87	30.09	240	47.18	56.96
73	51.71	45.9	241	56.8	55.37
74	54.75	56.01	242	44.63	51.17
75	50.91	51.62	243	44.85	43.59
76	52.97	48.54	244	50.99	52.53
77	50.49	49.04	245	44.82	42.6
78	54.07	52.81	246	46.8	51.79
79	49.07	51.27	247	32.13	32.16
80	56.38	56.35	248	50.4	57.25
81	51.65	47.83	249	47.71	56.57
82	51.55	26.14	250	41.9	49.94
83	49.05	34.6	251	53.23	52.07
84	49.6	52.71	252	46.41	55.8
85	53.15	46.7	253	56.45	58.76
86	51.72	54.91	254	49.62	49.88
87	50	51.55	255	44.03	40.16
88	49.89	57.55	256	45.32	39.72
89	43.29	50.7	257	50.51	49.7
90	46.95	53.9	258	55.23	53.88
91	47.54	42.2	259	53.7	58.03
92	57.49	57.9	260	40.98	44.2
93	54.73	50.85	261	39.75	41.15
94	55.64	54.13	262	44.49	57.52
95	54.23	54.97	263	49.61	52.21
96	49.01	37.89	264	48.36	50.93

97	52.85	45.47	265	44.19	51.63
98	53.22	55.1	266	49.17	59.33
99	52.01	52.79	267	55.01	39.77
100	51.59	53.71	268	50.71	45.93
101	36.55	35.7	269	52.35	51.43
102	43.33	32.75	270	54.08	49.81
103	44.32	34.68	271	52.34	53.85
104	51.43	51.1	272	46.57	53.6
105	53.81	52.9	273	39.55	41.32
106	51.92	55.17	274	51.89	48.43
107	50.87	49.37	275	51.61	55.37
108	49.15	54.99	276	53.54	46.75
109	47.02	46.16	277	30.15	31.47
110	49	42.5	278	46.93	45.72
111	39.66	38.35	279	48.98	33.69
112	54.73	36.15	280	36.23	51.42
113	37.97	48.37	281	43.93	41.73
114	51.27	57.39	282	48.01	48.79
115	55.51	57.48	283	42.06	41.86
116	42.77	22.15	284	48.39	51.42
117	43.17	57.04	285	45.45	46.12
118	52.75	44.93	286	53.59	41.99
119	51.37	51.07	287	50.25	54.07
120	47.41	53.72	288	50.69	52.01
121	48.1	51.89	289	56.95	57.66
122	49.25	57.37	290	46.48	51.75
123	53.12	48.33	291	54.93	59.77
124	52.5	57.45	292	53.77	53.23
125	48.79	43.53	293	56.18	54.85
126	48.45	51.47	294	54.28	55.56
127	56.68	60.24	295	49.49	48.99
128	53.3	51.43	296	48.3	49.34
129	55.8	56.48	297	48.72	52.96
130	54.41	51.37	298	53.73	53.87
131	57.68	54.55	299	50.9	44.15
132	53.65	55.45	300	52.57	44.2
133	51.91	54.55	301	50.25	47.67
134	51.6	59.65	302	52.51	47.46
135	57.89	57.83	303	49.75	46.28
136	52.79	54.83	304	49.57	39.32
137	52.64	52.43	305	54.61	51.82
138	44.48	33.29	306	52.38	56.91
139	54.86	66.36	307	53.13	56.36
140	46.74	16.25	308	55.45	60.75
141	45.63	57.39	309	56.54	60.04
142	53.07	58	310	52.21	51.51
143	51.87	44.09	311	46.38	47.81
144	43.75	63.4	312	47.71	44.86
145	50.95	54.92	313	49.96	55.37
146	53.09	49.97	314	42.99	48.01

147	59.16	59.85	315	43.83	49.39
148	63.29	60.67	316	47.63	48.57
149	56.95	56.69	317	54.48	55.68
150	49.93	56.29	318	46.89	38.02
151	51.81	58.65	319	46.29	51.43
152	44.33	36.55	320	52.77	55.76
153	48.55	51.99	321	53.14	42.14
154	52.62	56.49	322	49.07	49.37
155	48.87	56.37	323	54.91	48.75
156	51.26	53.53	324	55.83	55.52
157	39.57	45.44	325	51.15	49.91
158	48.57	56.96	326	56.63	47.45
159	50.87	59.57	327	48.13	41.72
160	53.1	52.4	328	45.4	42.77
161	52.15	55.25	329	50.5	52.25
162	57.44	58.61	330	61.34	55.76
163	55.09	54.45	331	55.37	52.09
164	51.77	36.24	332	49.45	47.36
165	59.04	59.23	333	58.77	58.31
166	48.24	56.09	334	49.26	55.03
167	50.23	47.02	335	58.47	54.61
168	47.97	55.26			

CK, control; DS, drought stress.
